# High rates of unsuccessful transfer to adult care among young adults with juvenile idiopathic arthritis

**DOI:** 10.1186/1546-0096-8-2

**Published:** 2010-01-11

**Authors:** Elizabeth Hazel, Xun Zhang, Ciarán M Duffy, Sarah Campillo

**Affiliations:** 1 Department of Medicine, McGill University, Montreal, Canada; 2 Department of Biostatistics, McGill University, Montreal, Canada; 3 Department of Pediatrics, McGill University, Montreal, Canada

## Abstract

**Background:**

This study aimed to describe the proportion of patients with juvenile idiopathic arthritis (JIA) who had experienced an unsuccessful transfer from a pediatric rheumatology team to an adult rheumatologist and to compare the characteristics of those who achieved successful transfer to those who did not.

**Methods:**

We conducted a systematic chart review of all patients with JIA who attended their final Montreal Children's Hospital JIA clinic appointment between 1992 and 2005. We tracked these patients for the two years after transfer to an adult rheumatologist. We then compared characteristics of patients with successful and unsuccessful transfers of care. Variables pertaining to disease characteristics, disease severity and psychosocial factors were examined. Univariate analyses were performed to determine if any single factor was associated with the outcome of unsuccessful transfer of care.

**Results:**

52% of patients fulfilled our criteria for unsuccessful transfer. Of the variables tested, an active joint count (AJC) of zero at last visit was associated with the outcome of unsuccessful transfer (OR = 2.67 (CI 1.16-6.16; p = 0.0199)).

**Conclusions:**

Despite the presence of a coordinated process of transfer from pediatric to adult health care for the majority of the patients in this study, there was a high rate of unsuccessful transfer and/or sustained follow up which is disheartening. We found that patients with less active disease at the time of transfer, as indicated by a lower AJC, were more likely to be lost to follow up. Recent literature suggests that even in the least severe categories of JIA, 50% of patients persist with active disease into adulthood. Thus educating all JIA patients about the possibility of disease flare in adulthood may improve their adherence to recommendations for sustained follow-up in the adult milieu. This may lead to improvement of longitudinal outcomes for all JIA patients.

## Background

In the past, the care of adults with Juvenile Idiopathic Arthritis (JIA) has been overlooked. JIA was once thought of a disease of childhood that "burnt out" by adulthood. Numerous studies have now confirmed that the majority of young people suffering from JIA will have persistent disease into adulthood. A comprehensive Canadian study showed that 58% of adult patients with Juvenile Rheumatoid Arthritis (JRA) did not meet criteria for disease remission [1]. Those patients with pauciarticular JRA had the best outcomes, with 47% achieving remission while they found that polyarticular rheumatoid factor (RF) positive patients had an "essentially unremitting" course. In addition, many more patients continue to suffer from long term consequences, beyond the physical disability associated with their disease. These include psychosocial and socioeconomic factors which contribute to a diminished quality of life [2]. JIA carries a high burden of disease in many adults and it is, therefore, of the utmost importance to ensure a smooth transfer from the pediatric to the adult health care teams.

Recently, we have seen an increased interest in the area of transitional care in rheumatology. There is a burgeoning literature focused on improving the transfer of care of young adults with rheumatic diseases by implementing a programme of transitional care for adolescents [3]. However, there is no literature to date, which has studied the rates of unsuccessful transfer in the young adult population with JIA. Similarly, there has been very little objective research aimed at identifying patient and disease specific characteristics that may impede successful transfer.

A number of factors have been identified subjectively as barriers to the smooth transition of care from pediatric to adult rheumatologists. A postal survey conducted by Shaw in 2004 reflected the feeling among respondents that inadequate resources, problems of institutional support, poor inter/intra agency coordination and lack of physician education were the major barriers to providing optimal transitional care [4]. These health professionals identified risk factors for transitional difficulties including family dynamics, adolescent intrapersonal characteristics and disease severity. Despite these perceptions on transitional care, objective research aimed at identifying patient and disease specific characteristics that may impede successful transfer has never been performed in the JIA population.

The Montreal Children's Hospital is part of the McGill University Health Center (MCH-MUHC) and is a tertiary care teaching hospital. The weekly JIA clinic cares for patients from the greater Montreal area, and beyond. Ideally, the transfer to adult care is completed prior to, or during the patient's 19^th ^year. Most of these patients are transferred to an adult rheumatologist within the McGill network, but some are transferred to different adult rheumatologists based on language preference, location or other issues. Currently, a transfer letter is routinely sent to the adult rheumatologist. The young adult is given the responsibility of booking the initial appointment but follow-up with the pediatric nursing team is maintained until transfer is completed. However, it should be noted that the nursing support evolved over the time course of this study.

We conducted a descriptive, retrospective study of patients with JIA who attended the MCH JIA clinic and who were subsequently transferred to the care of adult rheumatologists between 1992-2005. The objectives of this study were two-fold: firstly, to describe the proportion of young adults with JIA who had experienced an unsuccessful transfer from a pediatric rheumatology team to an adult rheumatologist and secondly, to identify patient characteristics associated with unsuccessful transfer of care. Although we recognized that the process of transition begins much earlier in the MCH JIA clinic, only transfer of care was evaluated in this study.

The definition of unsuccessful transfer was operationally defined as failure to make initial contact with an adult rheumatologist, or failure to continue to follow-up with an adult rheumatologist two years after transfer (no contact for a one year period after the last scheduled appointment).

## Methods

A systemic chart review of all patients with a diagnosis of JIA who had attended the MCH JIA clinic between 1992 and 2005 was conducted after scientific approval for quality of care research was obtained. Children with JIA have a parallel JIA chart, in addition to the hospital chart. The JIA charts are kept in the Division of Rheumatology. Once a patient is no longer actively followed by the rheumatology team, his/her JIA chart is moved to a separate archive file. All archived charts were reviewed, and the hospital charts were reviewed as needed. Patients who were discharged from the service or lost to follow-up before the age of 18 and those in whom follow-up care was not deemed necessary were excluded from the analyses.

We then tracked the follow-up these patients received in the adult milieu for the two years after transfer. Once the name of the adult rheumatologist was identified in the transfer letter, or the last clinic note, his/her office was contacted for permission for a chart review to be conducted. The chart was then reviewed for the time period of two years after transfer. In cases where it was inconvenient to directly review the adult chart, the information was obtained over the phone from the adult rheumatologist.

If a patient never made contact with the identified adult rheumatologist or was lost to follow-up at two years following transfer, then this patient was deemed to have had an unsuccessful transfer. This definition of unsuccessful transfer of care is similar to the definition which has been used in another study of the transfer of care in patients with a chronic disease [5].

The characteristics of patients with and without successful transfer of care were compared. Variables pertaining to demographic and disease characteristics (gender, category of JIA, age at diagnosis and presence/absence of uveitis), disease severity (use of disease modifying agents of rheumatic disease (DMARDs) and active joint count) and psychosocial factors (educational attainment) were examined. DMARDs included azathioprine, cyclosporine, etanercept, gold, hydroxychloroquine, infliximab, methotrexate, and sulfasalazine. Category of JIA was documented as per the International League Against Rheumatism (ILAR) classification [6]. In some cases, we reassigned a diagnosis based on laboratory and clinical findings from the clinic chart. Patients were classified as having oligo-arthritis, polyarthritis rheumatoid factor (RF) negative, polyarthritis rheumatoid factor positive, psoriatic arthritis, systemic arthritis, enthesitis-related arthritis or undifferentiated arthritis. Use of DMARDs was defined as to whether or not the patient had ever used any DMARDs. The active joint count (AJC) documented at the clinic visit closest to the patient's 18^th ^birthday was recorded. AJC was categorized as a binary variable, zero versus one or more active joints (the AJC is routinely recorded in the JIA chart at every clinic visit, and is defined as the presence of an effusion or loss of movement with pain, heat or tenderness). The patient's educational attainment at the time of transfer was noted. If they had not completed secondary school or were more than one year behind their peers, they were considered to have a delayed educational attainment.

### Statistical Analysis

Descriptive statistics were employed including means and standard deviations for continuous variables; frequencies and proportions were used for categorical variables. Univariate analyses were performed in order to identify associations between the independent variables and the outcome of unsuccessful transfer of care. Given the small number of patients with RF positive polyarthritis, this category was combined with RF negative polyarthritis for the sake of the analysis. The chi-squared test was employed for categorical variables whereas the unpaired t-test was used for continuous variables. The level of significance was set at a p value of less than 0.05. Odds ratios (OR) were calculated for binary categorical variables although we acknowledge that this project was not a true case-control study.

## Results

**Figure 1:**
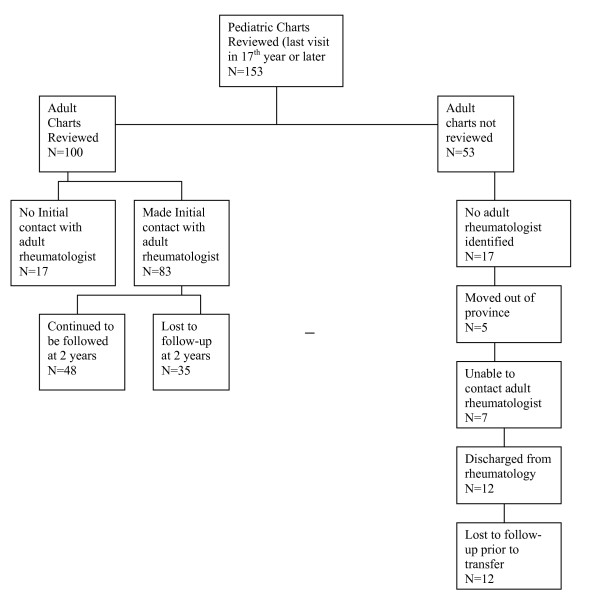
**Participant Flow Diagram**.

For the descriptive study, the charts of 153 patients with JIA were reviewed. From these, 53 patients were excluded from the analysis for various reasons. There was inadequate information in the chart to identify an adult rheumatologist in 17 cases. Five patients were transferred to rheumatologists living outside of the province of Quebec. The adult rheumatologist could not be contacted for another 7 patients. Twelve patients were discharged from the clinic, with their pediatric rheumatologist deeming that transfer to an adult rheumatologist was not indicated. Twelve patients were lost to follow-up prior to transfer. In these cases, the patients did not attend consecutive scheduled JIA clinics, despite follow-up telephone calls by the pediatric team (See Figure 1).

The study population was 68% female with an average age of diagnosis of JIA of 9.84 years. All subtypes of JIA were represented. The mean AJC at the final visit was 1.15 joints, and 49.4% had ever used DMARDs. For 90% of the patients studied, their education was on track with their peers (See Table 1).

**Table 1:** Baseline Characteristics

	Baseline Characteristics
JIA category	
Systemic Arthritis	7 (7%)
Oligoarthritis	21 (21%)
Polyarthritis RF+	6 (6%)
Polyarthritis RF-	17 (17%)
Psoriatic	7 (7%)
ERA	30(30%)
Undifferentiated	12 (12%)

Age at diagnosis (mean in years +/- 1 std dev)	9.84 +/- 4.81

Female	68%

AJC = 0	63 (63%)
AJC > = 1	37 (37%)

DMARD use (ever)	49.4%

Education on track *	90%

*9 missing values

DMARD- Disease modifying Agent of Rheumatic Disease

AJC- Active Joint Count

n- number

%- per cent

Std dev- standard deviation

Of the 100 adult charts reviewed, 52 patients fit our criteria for unsuccessful transfer. Of these, 17 made no initial contact with the appointed adult rheumatologist and 35 were lost to follow-up at two years after transfer. Therefore, 52% of our patients had an unsuccessful transfer from pediatric to adult care (See Figure 1).

A comparison of the characteristics of patients with successful transfer of care and unsuccessful transfer of care was conducted (See Table 2). From the univariate analyses, a low AJC identified at the last pediatric rheumatology clinic visit was significantly associated with unsuccessful transfer of care (p value 0.02;OR 2.67). Additionally, boys trended toward higher risk for unsuccessful transfer of care (p value 0.08;OR 2.15). All other variables were not found to be associated with the outcome of interest.

**Table 2:** Comparison of characteristics of patients with successful and unsuccessful transfer of care

	Successful Transfer (n = 48)	Unsuccessful transfer (n = 52)	P-values
JIA category			0.18
Systemic Arthritis	6	1	
ERA	12	18	
Oligoarthritis	7	14	
Polyarthritis	13	10	
Psoriatic	4	3	
Undifferentiated	6	6	

Uveitis (n, %)	8 (17.0%)	9 (17.0%)	0.99

Age at diagnosis (mean, year)	9.27	10.35	0.26

Use of DMARDs (n, %)	27 (57.4%)	31 (58.5%)	0.92

AJC (n, %)			0.02
0 joints	24(51.1%)	39 (73.6%)	
> = 1 joints	23 (48.9%)	14 (26.4%)	

Gender			0.08
Female (n, %)	36 (76.6%)	32 (60.4%)	
Male (n, %)	11 (23.4%)	21 (39.6%)	

Educational attainment			0.25
Education on track with peers* (n, %)	28/43 (65.1%)	38/48 (79.2%)	
Not on track	15/43 (34.9%)	10/48 (20.8%)	

*Missing information in 9 subjects

DMARD- Disease modifying Agent of Rheumatic Disease

AJC- Active Joint Count

n- number

%- per cent

## Discussion

This is the first study to describe the prevalence of unsuccessful transfer of care in a juvenile arthritis population. We found that over half of young patients transferred to an adult rheumatologist had inadequate follow-up for their arthritis over the ensuing two years. This finding is surprising, given that our patient population probably represented the more severe end of the spectrum of JIA. This was reflected by the over representation of the types of JIA which are more likely to persist into adulthood, or lead to long-term disability. In addition, there likely was a referral bias because the Montreal Children's Hospital is a tertiary care hospital and most patients were referred to adult rheumatologists affiliated with University hospitals.

The long-term care of adults with JIA presents a number of challenges which are unique to this patient population [7]. The long-term consequences of JIA encompass both disease specific sequelae and decreased social and economic performance which may contribute to diminished quality of life [8]. In many cases, coping strategies are not fully developed before adolescence and the formative years must be negotiated with a chronic disease in evolution [2].

There are a number of studies, which have now confirmed that a large percentage of young people suffering from JIA will continue to have active disease into adulthood. In a review of this topic by Kiem Oen, she concluded that JIA often extends into adulthood. She found that most remissions will occur within the first 5 years and that the probability of remission decreases progressively after that [1]. In fact, the majority of adult patients studied did not meet criteria for disease remission. Similarly, a long-term follow-up study by Zak and Pedersen showed evidence for increasing disability and residua with increasing follow-up time. Moreover, over half of the patients studied reported arthritis related pain, including a proportion who fit criteria for disease remission [9].

Our proportion of unsuccessful transfer of 52% is strikingly similar to that described in a population of patients with congenital heart disease, with fewer than 50% having appropriate follow-up in the adult milieu [5]. The patients studied were at high risk of arrhythmias and re-operation. Successful transfer of care was significantly related to the presence of documented recommendations and to patient beliefs that his/her care should continue into adulthood in a specialized center. Patients who had had more cardiac surgeries, those who had refrained from abusing substances, and those who had attended pediatric appointments alone were also more likely to be followed appropriately in the adult centers.

A similar rate of unsuccessful transfer was described in a study of patients with type 1 diabetes [10]. In this study, the baseline drop out rate in the adult centers was 40%. The authors of this study were able to improve this rate to 11% by providing a cohort of young adults support in navigating in the adult health care system in the transition period between pediatric and adult care.

Every effort should be made to ensure that young adults with JIA have timely access to a rheumatologist in the event of a disease flare, in order to minimize their disease burden. We found an association between a low AJC at last pediatric clinic visit and an almost three-fold risk of unsuccessful transfer. This group of young adults with relatively inactive disease should be educated about the importance of ongoing follow-up in the adult milieu given the high possibility of active disease into adulthood.

The process of transitioning children to adult care should begin long before the time of transfer. The Canadian Pediatric Society's position statement, published in 2007 emphasizes the differences between the family focused pediatric care and the patient focused adult milieu [11]. This different "culture" may play a role in unsuccessful transfers of care. The report highlighted the fact that many of the barriers to successful transfer are the same across a wide variety of chronic diseases [12-15]. Health care transition counseling with enhanced social support and self-management strategies may improve outcomes [16,17].

Our study did have limitations. The retrospective cohort design of this study has some notable disadvantages. We were unable to track down a large number of patients in the cohort and we relied heavily on documentation in the charts to ascertain the transition plan. It is possible that lack of documentation led us to over-estimate the rate of unsuccessful transfer. For example, patients lost to follow-up or discharged from the JIA clinic may have sought follow-up in the adult milieu on their own. However, these transfers would have been suboptimal at best, with no transfer letter sent to the adult rheumatologist. We were also unable to evaluate several potentially important barriers to successful transfer of care, identified by Shaw [4]. Those variables pertaining to adherence to treatment, pain, functional status, number of independent visits, psychosocial functioning, support system and economic factors should be evaluated prospectively. Additionally, the small study size may have impaired our ability to identify significant risk factors for unsuccessful transfer.

The proportion of subtypes of JIA differs in our study to the numbers described in the literature [18]. There is a marked under-representation of oligoarthritis (18% compared to 27-56%) and an over-representation of enthesitis-related arthritis (24% compared to 3-11%), while the other disease subtypes are appropriately represented. This pattern may reflect the forms of juvenile arthritis which are more or less likely to persist into adulthood. We may have captured some patients who were labeled as ERA but later evolved to become inflammatory bowel disease-related arthropathy. Additionally, this population is drawn from a tertiary care centre which may skew the representation towards more serious cases of juvenile arthritis. It is interesting to note that, although not statistically significant, there was a trend towards less successful transfer of care in certain JIA categories (oligoarthritis, ERA) and successful transfer in systemic arthritis. The possible association between category of JIA and unsuccessful transfer of care should be addressed in future studies.

We found that those patients with less active disease, with an AJC of zero at the last clinic visit prior to transfer, were almost three times more likely to have an unsuccessful transfer to adult care. Alerting the pediatric team to the importance of educating the patients with less severe disease about the possibility of active disease into adulthood, and the importance of having follow-up with an adult rheumatologist is important. In our study, we noted a trend for young men to have unsuccessful transfers. Targeting this group may also be important to improving the follow-up of these patients.

The number of patients in each category of JIA was probably too small to allow for detection of differences between categories. Disease duration was perceived to be a risk factor for transitional difficulties in the study by Shaw. In our study, age of onset did not predict unsuccessful transfer of care; we used this variable as a surrogate for disease duration as most patients transferred in their nineteenth year. Because the treatment practices changed over the course of the time period covered by the study, use of DMARDs and biologic agents at the time of the transfer was not evaluated. Whether the patient had ever used DMARDs was evaluated as a marker of disease severity, but it did not significantly predict the outcome of unsuccessful transfer of care.

In conclusion, we found a dishearteningly high rate of unsuccessful transfer for pediatric to adult rheumatology care. Since this study was terminated, a dedicated young adult rheumatic disease clinic has been established within the McGill network. It is our hope that this clinic will be able to address some of the barriers to successful transfer of care. A follow-up study to test this hypothesis is planned.

## Competing interests

The authors declare that they have no competing interests.

## Authors' contributions

EH conceived the study design, performed the chart reviews and drafted the manuscript, XZ performed the statistical analysis, CD participated in the study design and SC participated in the study design and manuscript production. All authors read and approved the final manuscript.
